# Multiple gene-drug prediction tool reveals Rosiglitazone based treatment pathway for non-segmental vitiligo

**DOI:** 10.1007/s10753-023-01937-9

**Published:** 2023-12-30

**Authors:** Sijia Zhao, Xi Chen, Kuheli Dutta, Jia Chen, Juan Wang, Qian Zhang, Hong Jia, Jianfang Sun, Yongxian Lai

**Affiliations:** 1 Department of dermatologic Surgery, Shanghai Skin Disease Hospital, School of Medicine, Tongji University, Shanghai, China; 2 Department of Dermatology, Allergology and Venereology, Universitätsklinikum Schleswig-Holstein, Lübeck, Schleswig-Holstein Germany; 3 School of Medicine, Shanghai University, Shanghai, China; 4 Department of Pathology, Institute of Dermatology, Chinese Academy of Medical Sciences and Peking Union Medical College, Nanjing, People’s Republic of China

**Keywords:** non-segmental vitiligo, rosiglitazone, PPAR pathway, melanogenesis, bioinformatics analysis

## Abstract

Vitiligo is a skin disease characterized by selective loss of melanocytes, which seriously affects the appearance and causes great psychological stress to patients. In this study, we performed a comprehensive analysis of two vitiligo microarray datasets from the GEO database using bioinformatics tools to identify 297 up-regulated mRNAs and 186 down-regulated mRNAs, revealing important roles for pathways related to melanin synthesis, tyrosine metabolism, and inflammatory factors, such as “PPAR signaling pathway”, “tyrosine metabolism”, “nonalcoholic fatty liver disease (NAFLD) pathway”, “melanogenesis”, and “IL-17 signaling pathway”. Combining the Search Tool for Interacting Chemicals (STITCH) database 5.0 and the drug-gene interaction database 3.0 (DGIdb), we identified that the PPAR-γ agonist rosiglitazone may promote melanin synthesis via EDNRB. Next, we investigated the mechanism of rosiglitazone and PPAR-γ pathway in promoting melanin production. Consistent with the results of bioinformatics analysis, the expression levels of PPAR-γ, EDNRB, and TYR were significantly reduced in human non-segmental vitiligo skin along with the reduction of MITF, a key gene for epidermal melanogenesis. Meanwhile, rosiglitazone increased melanin synthesis capacity in melanocytes and zebrafish by activating PPAR-γ and upregulating TYR, TYRP-1, and TYRP-2. Conversely, treatment of melanocytes with the PPAR-γ antagonist GW resulted in inhibition of melanin synthesis and expression of melanin-related factors. At the same time, simultaneous treatment of rosiglitazone with GW reversed the inhibitory effect of GW on melanin synthesis. In this study, we identified that rosiglitazone, an important insulin sensitizer, promotes melanin synthesis in melanocytes by increasing PPAR-γ activity and upregulating the expression levels of EDNRB and TYR. These findings may provide new ideas for exploring the pathogenesis and potential therapeutic targets of non-segmental vitiligo.

**Supplementary Information:**

The online version contains supplementary material available at 10.1007/s10753-023-01937-9.

## INTRODUCTION

Vitiligo is a depigmentation disease characterized by the destruction of epidermal melanocytes, clinically manifested as hypopigmented or depigmented patches of skin and mucous membranes [1]. Generally speaking, the incidence of vitiligo is about 0.5–1% and may be higher in localized areas and ethnic groups [2]. Although vitiligo is not life-threatening, the lesions often occur in exposed areas, especially the face and arms, imposing a huge psychological and economic burden on patients.

The pathogenesis of vitiligo is believed to be associated with multiple factors including genetics, autoimmunity, oxidative stress, melanocyte apoptosis, and neurological mechanisms [3–5]. Previous studies have demonstrated that oxidative stress-induced damage to melanocytes within vitiligo lesions results in the release of exosomes containing melanocyte-specific antigens. These exosomes subsequently stimulate dendritic cells to differentiate into mature antigen-presenting cells, ultimately leading to the differentiation of CD4+ T cells into Th1 or Th17 lymphocytes that secrete various cytokines including interferon-γ (IFN-γ), tumor necrosis factor-alpha (TNF-α), and interleukin-17 (IL-17). These cytokines further damage melanocytes and activate B lymphocytes to produce antibodies against autoantigens, such as tyrosinase, tyrosin hydroxylase, and Sox10 [6–9]. Simultaneously, CD8+ T cells are activated and exert their destructive effect on melanocytes through three distinct mechanisms: perforin/granzyme B-mediated cytotoxicity, TNF-α production, or via CTL activation through Fas/FasL interaction. This results in a reduction in the number and function of melanocytes, ultimately leading to skin depigmentation [10].

Therefore, the treatment of vitiligo is divided into two stages. The initial focus should be on correcting the abnormal immune response responsible for the destructive effect on melanocytes in progressive vitiligo. Subsequently, the focus shifts to promoting the proliferation of residual melanocytes in the affected area and surrounding regions, improving their melanin production ability, and stimulating melanocytes to induce repigmentation of affected areas during the stable stage of vitiligo [11]. For vitiligo patients in progressive stages, the application of corticosteroids reduces the gene expression of a large number of cytokines such as TNF-α and interferon-γ, thereby inhibiting the activation of cytotoxic T lymphocytes and reducing the response of B cells to autoantigens, as well as stimulating the production of pigment by melanocytes in the affected kind [12]. Topical calcium-regulated neurophosphatase inhibitors exhibit immunomodulatory effects by inhibiting the production of cytokines such as IL-2 and IFN by cytotoxic T cells, thereby controlling the progression of vitiligo [13, 14]. For vitiligo patients in stable stages, phototherapy is a crucial treatment option, especially for patients with more than 10% body surface area involvement. Following the determination of the minimum phototoxic dose, the administration of a light source comprising broadband ultraviolet radiation A (UVA, 320–380 nm) along with oral or topical psoralen can promote melanocyte growth by activating hair follicle melanocytes and releasing keratin-forming cell growth factors [15]. NB-UVB has been shown to promote the pigmentation of vitiligo lesions by inducing tyrosinase production and increasing the expression of HMB45 on the surface of melanosomes. Furthermore, prostaglandin analogs can stimulate tyrosinase production, upregulate melanocyte proliferation, and increase skin pigmentation [13].

Despite the availability of numerous treatment options, some patients remain resistant to conventional treatments or are associated with adverse effects such as damage to the skin barrier caused by the topical application of glucocorticoids or an increased risk of skin cancer with photochemotherapy. Therefore, increasing the understanding of the inflammatory pathways in vitiligo and searching for new drugs that can promote the proliferative activity of melanocytes remains important in this field. Nowadays, with high-throughput sequencing and bioinformatics analysis methods, gene chips have become an important tool to explore disease mechatnisms, allowing large-scale, efficient access to gene expression data for a variety of diseases, such as cancer, metabolic diseases, and immune diseases [16, 17]. Through comprehensive analysis of the vitiligo dataset in the GEO database, several studies have identified genes and pathways that influence vitiligo onset and progression, such as the WNT cell signaling pathway [18], the SCF-KIT signaling pathway, and oxidative stress [19], providing important insights into the pathogenesis of vitiligo. However, more studies are needed to clarify the pathogenesis of vitiligo and find more potential therapeutic drugs.

In this study, the vitiligo microarray datasets (GSE75819 and GSE65127) from the GEO database were used for comprehensive bioinformatics analysis. Using Gene Ontology (GO), Kyoto Encyclopedia of Genes, and Genomes (KEGG) analysis, we identified important roles for pathways related to melanin synthesis, tyrosine metabolism, and inflammatory factors. Subsequently, using protein interaction analysis, we screened for pivotal genes in vitiligo, of which the peroxisome proliferator-activated receptor signaling pathway (PPAR pathway) is strongly associated with vitiligo pathogenesis. Further, drug-gene interaction analysis of these genes suggested that an agonist of PPAR-γ, rosiglitazone, may activate the EDNRB gene and affect pigment synthesis. Based on this, we proposed the hypothesis that PPAR-γ expression is impaired in vitiligo patients, and rosiglitazone may promote pigmentation through upregulation of PPAR-γ and EDNRB genes. To verify these hypotheses, in the next study, we examined the expression levels of PPAR-γ and melanin synthesis-related factors in the skin of non-segmental vitiligo patients. In addition, the possible pro-melanin synthesis role of rosiglitazone was further investigated *in vitro* and in zebrafish. Through the above study, we hope to further explore the pathogenesis of non-segmental vitiligo and clarify whether rosiglitazone, a PPAR-specific agonist, can promote melanocyte proliferation and pigmentation through up-regulation of PPAR pathways, providing reference ideas for the search of new potential vitiligo therapeutic agents.

## MATERIAL AND METHODS

### Study Design

**Fig. 1:**
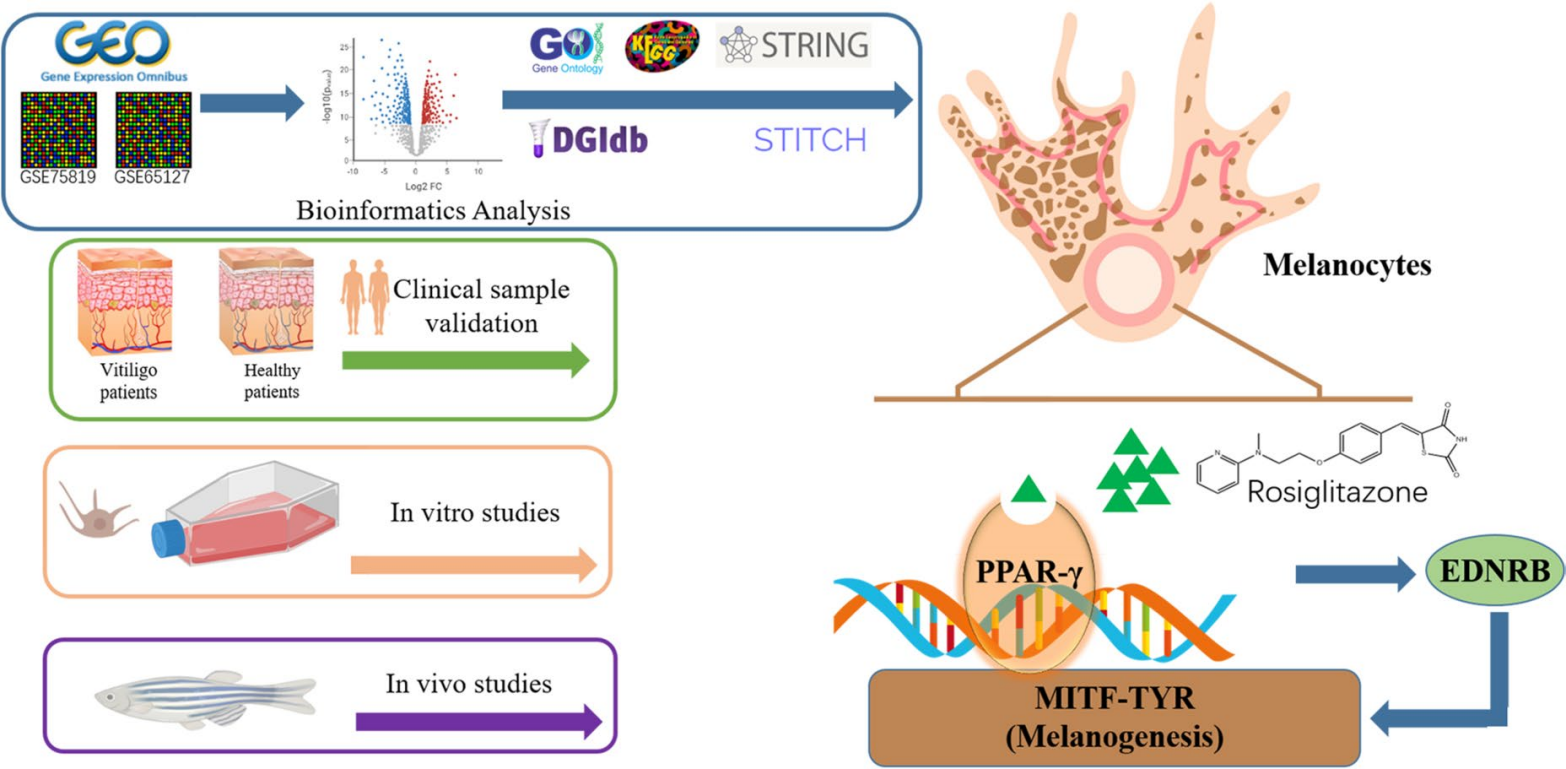
Summary of the effects of rosiglitazone on vitiligo.

The workflow diagram of this study is shown in Fig. 1. We first analyzed human vitiligo-related gene expression profiles derived from the NCBI-GEO database (https://www.ncbi.nlm.nih.gov/geo/) and identified the close association of the metabolism-related pathway PPAR-γ with the melanin synthesis pathway by GO and KEGG enrichment analysis. Subsequently, STITCH and DGIdb were used to explore compounds matching the potential gene expression profile. Based on these combined analytical data, we hypothesized that the agonist of PPAR-γ, rosiglitazone, may promote melanin synthesis by upregulating the expression level of EDNRB. To test this hypothesis, we determined that the expression level of PPAR-γ was positively correlated with impaired pigment synthesis in skin samples from non-segmental vitiligo patients and normal subjects. Finally, we verified the relationship between rosiglitazone and melanin synthesis through *in vivo* and *in vitro* experiments to determine its potential therapeutic effect on non-segmental vitiligo.

### Microarray Data Collection

The keywords “(vitiligo) and *Homo sapiens* [Organism]” were used to search the GEO database (https://www.ncbi.nlm.nih.gov/geo/) and a total of 14 datasets were obtained. Other inclusion criteria included that (a) the dataset was a genome-wide mRNA transcriptome data matrix; (b) the samples were derived from lesional and non-lesional skin tissue of vitiligo patients; (c) both raw and normalized datasets were acceptable. The exclusion criteria were (a) the samples were derived from melanocytes or blood; (b) the datasets were short of non-lesional skin samples of vitiligo patients; and (c) the datasets were of other species, such as mice. Ultimately, the lesional and non-lesional samples from the microarray datasets GSE75819 [19] and GSE65127 [18] were selected for comparison. The details of the datasets are shown in Table 1.

**Table 1:** Details of the GEO Vitiligo Data

Reference	Sample	GEO	Platform	Healthy	lesional	non-lesional	Peri-lesional
Singh et al. (2017) [19]	Skin tissue	GSE75819	GPL6884	-	15	15	-
Regazzetti et al. (2015) [18]	Skin tissue	GSE65127	GPL570	10	10	10	10

### Data Preprocessing and DEGs Screening

Both the series matrix and platform annotation files were downloaded from the GEO database by the “GEO query” package in R language software (https://www.r-project.org,version 3.6.3). Log_2_ transformation was accomplished on each matrix and the gene probe IDs were converted to gene symbols using R language commands. In order to reduce the discrepancies between datasets, quantile normalization was applied by the “normalize Between Arrays” function of the “limma” package in R language (http://www.bioconductor.org). Upon normalization, the GSE75819 and GSE65127 were merged after eliminating the batch effect between the datasets by the “limma” package and “sva” package in R language. Next, differentially expressed genes(DEGs) between the lesional and non-lesional of vitiligo patients were identified by multiple linear regression using “limma” package. The cut-off criterion were adjusted *p*-value < 0.05 and |log2 fold change (FC)| > 1. Finally, the hierarchical clustering analysis of the top 100 DEGs sorted by fold change were performed by “ggplots” package.

### GO Functional Term and KEGG Pathway Enrichment Analyses

Currently, the Kyoto Encyclopedia of Genes and Genomes (KEGG) pathway analysis and the Gene Ontology (GO) analysis were two of the most commonly used functional enrichment analyses. To further explore the role of the DEGs in the pathogenesis of vitiligo, KEGG and GO analyses were performed using the Metascape online tool (http://metascape.org/).

### PPI Networks and Hub Modules analyses

In order to construct the protein–protein interactions (PPI) network of DEGs encoded proteins, all up-regulated and down-regulated DEGs were analyzed by the STRING database (http://www. String-db.org). In addition, the hub module and associated genes (MCODE module) of the PPI network were obtained using the CytoHubba plugin of the Cytoscape software 3.7.2 (https://cytoscape.org) [20].

### Gene-Drug Prediction

To predict the potential treatment targets of vitiligo, drug-gene interaction analysis was performed by the Search Tool for Interacting Chemicals (STITCH, http://stitch.embl.de/) database 5.0 and the drug-gene interaction database 3.0 (DGIdb, http://www.dgidb.org/). The Search Tool for Interacting Chemicals database 5.0 (STITCH, http://stitch.embl.de/) integrates a variety of information including metabolic pathways, crystal structures, and binding experiments, and establishes drug–target relationships based on phenotypic effects, text mining, and chemical structure. The database contains information on the interactions of over 68,000 different chemical substances (including 2200 drugs) and links them to 1.5 million genes and their interactions in the 373 genomes [21]. Similarly, the Drug-Gene Interaction Database (DGIdb, www.dgidb.org) is a web-based tool containing more than 40,000 genes and 10,000 drug-passes, which helps to find drug-gene interactions by integrating and analyzing resources from papers, databases, and networks to uncover lists of compounds with gene expression patterns highly relevant to the phenotype of interest and to identify novel pathways or genes involved in related biological processes [22, 23]. To date, these databases have been successfully used to identify promising compounds and combination therapies in several areas, such as bone and joint diseases [24], kidney disease [25], ovarian cancer [26], and liver cancer [27]. The DGIdb database integrates gene-drug interaction information from several databases, including DrugBank, PharmGKB, Chembl, Drug Target Commons, Therapeutic Target Database (TTD), and others.

### Clinical Samples and Cell Lines

Non-segmental vitiligo(*n* = 5)and unpaired normal(*n* = 5)skin tissue samples were acquired from patients undergoing surgical pathological biopsy at the Shanghai Skin Disease Hospital, School of Medicine from July to December 2022. The clinical characteristics of the non-segmental vitiligo patients and the healthy controls are shown in Table S1. No treatments were administered in these patients prior to surgery. All samples were obtained after written informed consent was provided, in accordance with the Code of Ethics of the World Medical Association (Declaration of Helsinki). And all samples were approved by the Ethics Committee of the Shanghai Skin Disease Hospital, School of Medicine and diagnosed according to histopathological evaluations.

Due to the difficulty of obtaining human primary melanocytes and the long transmission period, pigment-producing human or murine-derived melanoma cell lines are commonly used in pigment-related studies [28–31]. Therefore, the human-derived melanoma cell line, Mum-2C (Shanghai Zeye Biotechnology Co., Ltd), was used in this study. The cell line was cultured in DMEM (Gibico) medium supplemented with 10% fetal bovine serum (FBS; BI, Israel) at 37 °C in a humidified atmosphere containing 5% CO2.

### Immunofluorescence Staining Assay

Human skin biopsies were fixed in 4% paraformaldehyde and later embedded in paraffin, sectioned, and subjected to direct immunofluorescence staining. The primary antibodies were purchased from Abcam (Cambridge, UK), including anti-PPARy (ab178860, 1/250), anti-EDNRB (ab117529, 1/2000), anti-MITF (ab3201, 1 μg/ml), and anti-TYR antibody (ab180753, 1/200). Sections were blocked with 3% bovine serum albumin (Beyotime Biotechnology, Shanghai, China) for 30 min and incubated overnight at 4 °C with the primary antibody. Afterwards, the secondary antibody (Beyotime Biotechnology, Shanghai, China) was used at a dilution of 1:500 and was incubated for 50 min at room temperature. A fluorescent microscope (AIX81, Olympus, Tokyo, Japan) was used for image acquisition. Fluorescence intensity was quantified using image J image analysis software (National Institutes of Health, Bethesda, MA, USA).

Cells were fixed with 4% paraformaldehyde for 20 min at room temperature, permeabilized with 0.5% Triton X-100 for 20 min, and blocked with QuickBlock™ Blocking Buffer (Beyotime Biotechnology, No.P0260) for 30 min at room temperature. Cells were then incubated overnight at 4 °C with primary antibodies, including anti-TYR (1:500), TYRP-1 (1:300), TYRP-2 (1:300), MITF (1:500), and PPAR-γ (1:500), EDNRB (1/500). Next, cells were incubated with a secondary antibody (goat anti-rabbit Alexa Fluor 640 dye, 1:5000, Life Technologies) for 1 hour at 37 °C. Cell nuclei were stained with DAPI (Beyotime Biotechnology, No.C0003). Subsequently, a confocal laser scanning microscope (LSM 510) was used for the analysis.

### CCK8 Assay

The cells were placed in 96-well plates at a density of 5 × 10^3^ cells/well and cultured overnight. Then, the cells were cultured with a complete medium containing different concentrations of rosiglitazone (R2408, Sigma-Aldrich; Merck KGaA) or GW9662 (No.S2915, Selleck Chemicals, Houston, TX, USA) for 24, 48, and 72 h. After 24, 48 and 72 h, cells were incubated with CCK8 solution (10 μl/well, Beyotime Biotechnology, no. C0038) for 1 h at 37 °C. The absorbance was measured at OD 450 nm by a microplate reader (Thermo Fisher Scientific Inc.).

### Dopamine Oxidase Activity and Melanocyte Staining

Dopamine oxidase activity was measured according to the method of Chen et al [32]. Briefly, cells were cultured in 12-well plates and treatment with rosiglitazone (10 μM) and GW9662 (10 μM) for 48 h. After that, 1% Triton-X was added to the cell precipitate and placed at −80 °C for 30 min and at room temperature for 30 min, respectively. The supernatant obtained by centrifugation was then transferred to a 96-well plate. 0.1% _L_-DOPA was added and incubated at 37 °C for 24 h. Absorbance was measured at OD 430 nm (Thermo Fisher Scientific Inc.)

For melanocyte staining, after treated with rosiglitazone and GW9662 in 12-well plates, the cells were fixed with 4% paraformaldehyde and incubated for 4 h at 37 °C with 1 g/l of 0.1% _L_-DOPA solution [33]. A microscope was used for observation and image acquisition.

### Zebrafish Culture And Treatments

Zebrafish is a very widely used animal model for pigmentation studies [34–36]. In our research, wild-type, AB-strain zebrafish were purchased from the Chinese Zebrafish Resource Center (CZRC), and the fish were kept in a dark cycle with a 14:10 h photoperiod and fed alternate days with brine shrimp and commercial fish diets. All animal handling in this study was performed in strict accordance with the guidelines and regulations established by the Animal Ethics Committee of the Chinese Academy of Medical Sciences. Zebrafish were randomly divided into control group (DMSO), rosiglitazone 10 μM group (RGZ), GW9662 10 μM group (GW), and rosiglitazone 10 μM + GW9662 10 μM (RGZ + GW), with 50 fish in each group. Treated zebrafish will be used for histological staining and RT-PCR experiments.

### Zebrafish Staining

For zebrafish staining, after treatment with rosiglitazone and GW9662, 72 hpf zebrafish were washed with 1 × PBST and incubated with 4% paraformaldehyde at 4 °C overnight. Then, zebrafish samples were washed with PBST (PBS containing 0.1% Triton X100), dehydrated, and hydrated. Antigen repair was performed with Tris-HCL. Following the blocking procedure with 1% BSA + 2% goat serum + PBST, anti-PPAR-γ antibody was added for incubation, then the secondary antibody (goat anti-rabbit IgG secondary antibody, 1:250) was added overnight at 4 °C. Finally, the images were observed and acquired by fluorescence microscopy.

### Reverse Transcription-Quantitative PCR (RT-qPCR)

Total RNA was extracted using the E.Z.N.A.® Total RNA Kit (Omega Bio-tek, Inc.) and the ratio of 260/280 absorbance was calculated to assess RNA purity (NanoDrop; Thermo Fisher Scientific, Inc.). RNA was reverse transcribed to cDNA using PrimeScript™ RT Master Mix (Takara Bio, Otsu, Japan) according to the manufacturer’s protocol. Primers for TYR, TYRP-1, TYRP-2, MITF, PPAR-y, EDNRB, and β-actin are shown in Table S2. A total of 10 μl of reaction was prepared before amplification mixture, including 5 μl of 2 × AceQ® q-PCR SYBR Green Master Mix (Vazyme Biotech, Nanjing, Jiangsu, China), 0.5 μl of cDNA, and 0.2 μl of each primer and sterile distilled water. The q-PCR reactions were performed on a Light Cycler 480 system (Roche Applied Science, Mannheim, Germany) with the following thermal cycling conditions. Initial denaturation at 95 °C for 15 min, followed by 40 amplification cycles at 95 °C for 10 s and 58 °C for 30 s; and a final extension at 72 °C for 30 s. Relative mRNA expression was calculated by the 2^−ΔΔCq^ method.

### Western Blot Analysis

Cellular proteins were collected with RIPA lysis buffer and the concentrations were determined by BCA assay. Subsequently, proteins were loaded onto sodium dodecyl sulfate-polyacrylamide gels (SDS-PAGE) and transferred to polyvinylidene difluoride (PVDF) membranes. PVDF membranes were then incubated overnight at 4 °C with primary antibodies TYR (1:1000) and PPAR-γ (1:1000). Afterwards, the secondary antibody (1:2000) was incubated at room temperature for 1 h, and the membrane was exposed to enhanced chemiluminescence (Amersham Pharmacia, Piscataway, NJ, USA). Image J software was used to quantify band intensity. β-actin was used as a loading control.

### Statistical Analyses

Results were analyzed by Student’s t-test for two groups. *P* < 0.05 was considered to indicate a statistically significant difference. Results are presented as the mean ± SD. Graphs were prepared using GraphPad Prism (version 6.0 for Windows; GraphPad Software, Inc.).

## RESULTS

### Normalization and DEG Identification in Vitiligo

**Fig. 2:**
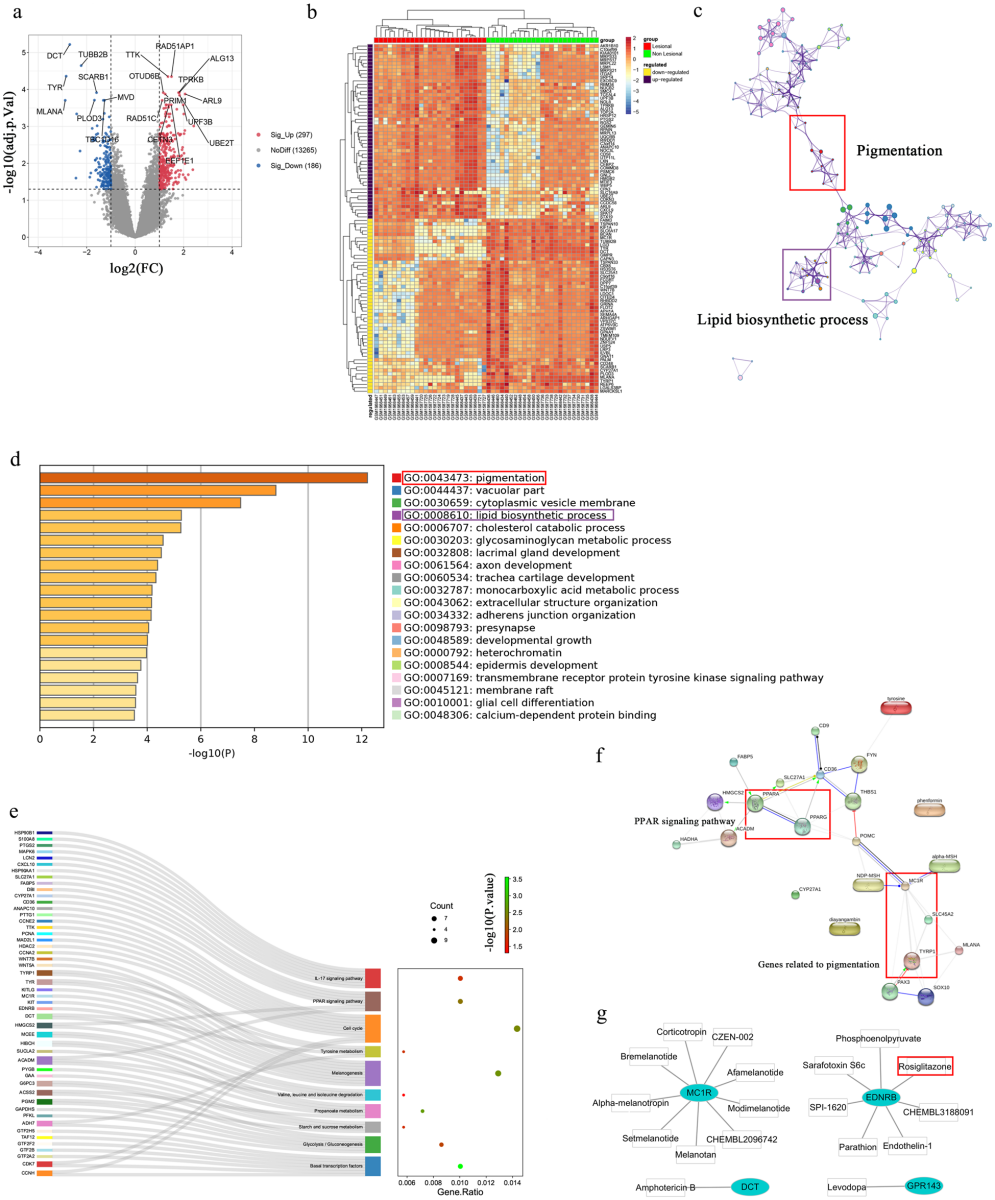
Differentially expressed genes (DEGs) between the vitiligo lesional skin group and non-lesional skin group among GEO-datasets. **a** Volcano plot of DEGs among GSE75819 and GSE65127 datasets. The red and blue spots represented relative upregulated and downregulated DEGs based on |fold change| > 1 and adjusted *p*-value < 0.05. **b** Cluster heatmap of the top 100 DEGs sorted by log fold change. **c** Network of GO term enrichment analysis (red frame are pigmentation-related genes; purple frame are lipid biosynthetic process–related genes). **d** GO terms enrichment analysis of DEGs is presented (each band representing an enriched term or pathway, colored according to a −log10 *p*-value). **e** Kyoto Encyclopedia of Genes and Genomes (KEGG) pathway enrichment by analysis of all differentially expressed genes (DEGs) (10 potentially vitiligo-relevant pathways and related genes). **f** Protein–protein interaction (PPI) network between PPAR signing pathway and pigmentation. **G** Gene-drug prediction of potential compounds.

To eliminate systematic errors and possible differences in the background, the sva package was used to normalize the datasets of GSE65127 and GSE75819 (Fig. S1a), A sum of 13,748 genes was used for differential analysis (Fig. S1b). Subsequently, the limma R package was used to screen for DEGs (adjusted *p* < 0.05 and |log FC| > 1). A total of 483 DEGs, including 297 up-regulated genes and 186 down-regulated genes, were identified in vitiligo skin compared to normal skin (Fig. 2a). The top 100 up/down-regulated DEGs sorted by log FC values are shown in the cluster heatmap (Fig. 2b).

### GO Function and KEGG Pathway Analysis

The Metascape database annotation tool was used for GO functional annotation and KEGG pathway analysis of DEGs. As shown in Fig. 2c and d, the GO functional annotation results showed that all differential genes were closely related to pigmentation and lipid biosynthesis. Respectively, in the Biological Processes (BP) category, the up-regulated DEGs were involved in “peptide biosynthesis” and “translation”; the down-regulated DEGs were mainly enriched in “lipid biosynthetic synthesis process” and “carbohydrate derivative biosynthesis process”. In the cellular components (CC) category, the up-regulated DEGs were significantly enriched in the “mitochondrial envelope” and “mitochondrial membrane”. The down-regulated DEGs were associated with “vacuole” and “lysosome”. In the molecular functions (MF) category, up-regulated DEGs were enriched in “structural molecular activity” and “structural components of ribosomes”. The down-regulated DEGs were associated with “oxidoreductase activity” and “actin binding” (Fig. S1c). Next, the KEGG pathway enrichment analysis was performed, and the results showed that all differential genes were mainly associated with “tyrosine metabolism”, “spliceosome”, “proteasome”, “PPAR signaling pathway”, “nonalcoholic fatty liver disease (NAFLD) pathway”, “melanogenesis”, “IL-17 signaling pathway”, “glycolysis/gluconeogenesis”, “galactose metabolism”, and “endocytogenesis” (Fig. 2e, Table S3).

### PPI Networks and Hub Modules Analyses

To further determine the protein–protein interactions of differential genes, the STRING database was used to construct a protein–protein interaction (PPI) network of DEGs (Fig. S1d). Notably, in this network, we found that the PPAR signaling pathway is closely associated with melanogenesis (Fig. 2f). In addition, we further analyzed the Hub genes in this network by Cytoscape software, where the top 10 Hub genes of upregulated DEGs including MAD2L1, CCNA2, PBK, MELK, KIF11, TTK, ASPM, RAD51AP1, CENPF, and TOP2A. The top 10 downregulated DEGs Hub genes include TYR, TRP-1, DCT (TRP-2), MLANA, MC1R, SLC45A2, SOX10, OCA2, KIT, and GPR143. Afterward, using the MCODE plugin in Cytoscape, we performed further functional analysis of the network. The KEGG results showed that among the 15 functional cluster modules screened from the PPI network, the up-regulated Hub genes were mainly related to “mitochondrial translation”, “oxidative phosphorylation”, and “spliceosome”, while the down-regulated Hub genes were related to “melanogenesis” and “tyrosine metabolism “ were closely related (Table 2).

**Table 2:** KEGG and GO Enrichment Terms Associated with the Hub Modules of DEGs

Module	Nodes	Edges	KEGG enrichment	GO enrichment
Up DEGs-1	58	821	Mitochondrial translation; mitochondrial translational termination; DNA replication	Mitochondrial translation; Mitochondrial translational termination; mitochondrial translational elongation
Up DEGs-2	56	598	Oxidative phosphorylation; Ribosome; Parkinson’s disease	Oxidative phosphorylation; Ribosome biogenesis; ATP metabolic process
Up DEGs-3	12	59	Spliceosome; Proteasome; RNA transport	RNA splicing; mRNA processing; mRNA transport
Down DEGs-1	12	55	Melanogenesis; Tyrosine	Pigmentation; developmental pigmentation; melanocyte differentiation

*KEGG* Kyoto Encyclopedia of Genes and Genomes, *GO* Gene Ontology, *DEGs* differentially expressed genes

### Gene-Drug Prediction

To identify potential compounds for vitiligo treatment, we performed drug-gene interaction analysis using a combination of DGIdb and The STITCH database on genes related to “melanogenesis”, “tyrosine metabolism”, and “MCODE core module”, including TYR, TRP-1, DCT (TRP-2), MLANA, MC1R, SLC45A2, SOX10, OCA2, KIT, GPR143, MLPH, EDNRB, and ADH7. Since the expression levels of these genes are down-regulated in vitiligo patients, we suggest that agonists of these genes may be potential drugs for vitiligo treatment. As shown in Table 3 and Fig. 2g, we found that the gene expression patterns of multiple drugs were significantly correlated with the down-regulated gene expression patterns in vitiligo patients, where rosiglitazone may increase melanogenesis by activating the EDNRB gene. In addition, rosiglitazone is also a specific agonist of PPAR-γ. This is identical to our KEGG enrichment results. Based on these results, we chose to further investigate the role of rosiglitazone in terms of melanin synthesis.

**Table 3:** STITCH and DGIdb Permuted Results Showing Compounds with Significant Negative Correlation with Genes Related to Melanin Synthesis

Gene	Compound name	Interaction	Database
DCT	Amphotericin	agonist	DGIdb
EDNRB	SPI-1620	agonist	DGIdb
EDNRB	Sarafotoxin S6c	agonist	DGIdb
EDNRB	Phosphoenolpyruvate	agonist	DGIdb
EDNRB	Parathion	agonist	DGIdb
EDNRB	Endothelin-1	agonist	DGIdb
EDNRB	CHEMBL3188091	agonist	DGIdb
EDNRB	Rosiglitazone	agonist	STITCH
GPR143	Levodopa	agonist	DGIdb
MC1R	Modimelanotide	agonist	DGIdb
MC1R	Corticotropin	agonist	DGIdb
MC1R	Setmelanotide	agonist	DGIdb
MC1R	Melanotan	agonist	STITCH
MC1R	CHEMBL2096742	agonist	DGIdb
MC1R	CZEN-002	agonist	STITCH
MC1R	Alpha-melanotropin	agonist	STITCH
MC1R	Bremelanotide	agonist	DGIdb
MC1R	Afamelanotide	agonist	DGIdb

### Impaired PPAR-γ and EDNRB Expression in Non-Segmental Vitiligo Lesions

**Fig. 3:**
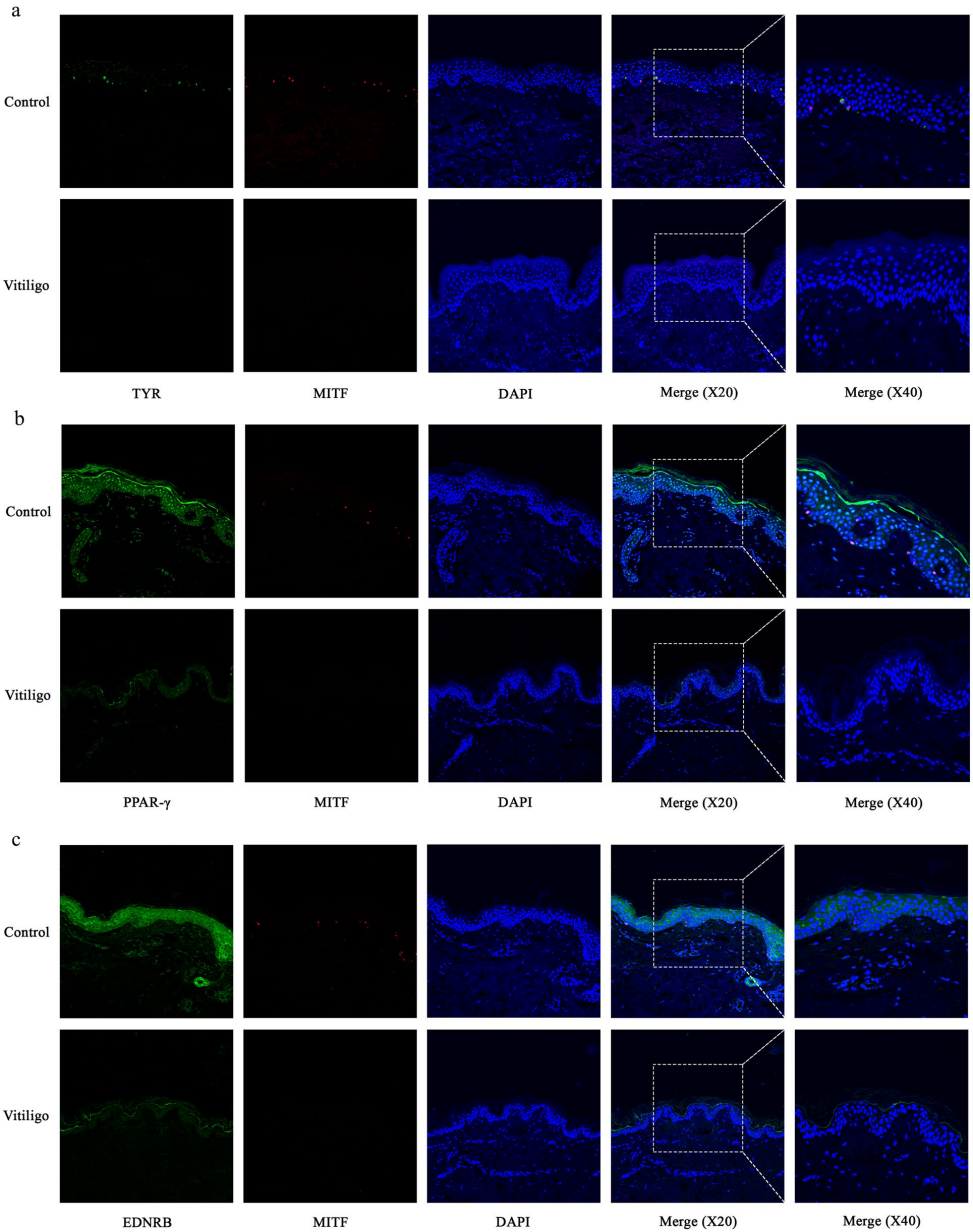
Melanocyte deficiency in vitiligo lesions is accompanied by impaired expression of PPAR-γ and EDNRB. **a** Representative images of the melanocytes in healthy skin samples (*n* = 5) and vitiligo lesions (*n* = 5) detected by immunofluorescence. Melanocytes were stained with antibodies to TYR (green) and MITF (red). Nuclei were counterstained with DAPI (blue). **b** Representative images of the expression of PPAR-γ (green) in healthy skin (*n* = 5) and vitiligo lesions (*n* = 5) detected using immunofluorescence. Melanocytes were stained with antibodies to MITF (red). Nuclei were counterstained with DAPI (blue). **c** Representative images of the expression of EDNRB (green) in healthy skin (*n* = 5) and vitiligo lesions (*n* = 5) detected using immunofluorescence. Melanocytes were stained with antibodies to MITF (red). Nuclei were counterstained with DAPI (blue).

By direct immunofluorescence, we examined the expression of PPAR-**γ** and melanogenesis-related genes EDNRB, MITF, and TYR in the skin tissues of non-segmental vitiligo patients (*n* = 5) and healthy people (*n* = 5). Previous studies have shown that MITF and TYR are specifically expressed in the nucleus and melanosomes of melanocytes, respectively. Therefore, MITF and TYR co-staining was used to specifically label melanocytes. The results showed that the expression of MITF and TYR was almost absent in non-segmental vitiligo lesions compared to healthy skin (Fig. 3a). Furthermore, we found that in healthy skin, PPAR-γ and EDNRB were significantly expressed in the nuclei of both keratinocytes and melanocytes, whereas in the epidermis of non-segmental vitiligo patients the expression of PPAR-γ and EDNRB was significantly reduced (Fig. 3b and c). These results suggest that defects in the PPAR pathway and reduced expression of EDNRB protein may be involved in the pathogenesis of non-segmental vitiligo.

### Effect of Rosiglitazone on Cell Viability

To determine the effect of different concentrations of rosiglitazone and GW9662 on cells, we assessed cell viability by CCK-8 to determine the optimal drug concentration. The results showed an increase in cell viability after treatment with 0.1 μM, 1 μM, and 10 μM Rosiglitazone for 48 h. However, a decrease in cell viability was observed after 72 h of treatment. In addition, to investigate the effect of PPAR-γ on melanogenesis, GW9662, an antagonist of PPAR-γ, was used in our study. The 10 μM GW9662 treatment for 48 h did not show a significant decrease in cellular viability. Therefore, in subsequent experiments, we treated cells with 10 μM rosiglitazone and 10 μM GW9662 for 48 h to avoid cytotoxicity (Fig. S2a).

### Rosiglitazone Promotes Melanogenesis Through the PPAR-γ Signaling Pathway *In Vitro*

**Fig. 4:**
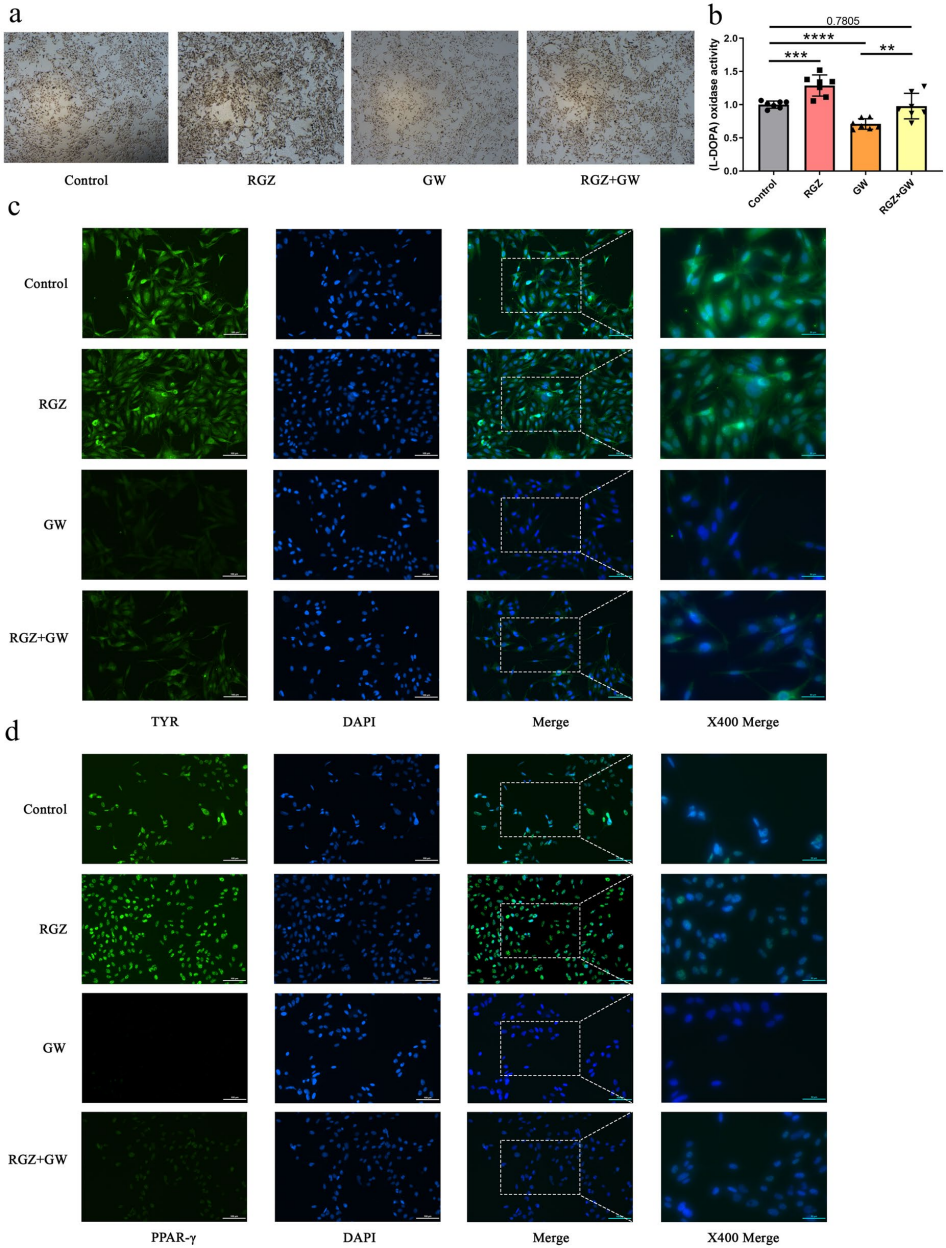
Effect of PPAR-γ signaling pathway on melanogenesis. **a**_L_-dopa staining of melanoma cells (Mum-2C) treated with rosiglitazone and GW9662 for 48 h. **b** Quantification of melanogenesis by _L_-dopa staining. **c** Immunofluorescence of TYR in melanoma cells for 48 h. **d** Immunofluorescence of PPAR-γ in melanoma cells for 48 h. Data represent mean ± 95% confidence interval (CI) ***p < 0.001; ****p < 0.0001; *n* = 7.

Next, we sought to elucidate the effect of rosiglitazone, an agonist of PPAR-γ, on melanogenesis through *in vitro* experiments. To this end, we used human melanoma to verify the effect of rosiglitazone on melanogenesis. First, after 48 h of treatment, _L_-dopa staining and quantification were used to detect melanogenesis levels. The results showed that rosiglitazone (10 μM) significantly increased melanogenesis levels. Conversely, GW6225 (10 μM), an inhibitor of PPAR-γ, significantly reduced melanin nodulation. Interestingly, as shown in Fig. 4a and b, rosiglitazone was effective in reversing the inhibitory effect of GW6225 on melanogenesis but did not show significant changes compared to the control group (*p* = 0.7805).

Subsequently, to further clarify the potential mechanism by which rosiglitazone increases melanocyte melanogenesis, RT-PCR and Western-blot were used to detect the expression levels of PPAR-γ and melanin synthesis-related factors, such as EDNRB, MITF, TYR, TRP-1, and TRP-2. Consistent with the results of bioinformatics analysis, the RT-PCR results showed that rosiglitazone significantly increased the expression levels of *ppar-γ* after the expression levels of *tyr*, *trp-1/2,* and *mitf* were also significantly increased. In contrast, after GW9662 inhibited *ppar-γ* expression, the expression levels of *trp-1/2* and *mitf* were significantly decreased, but *ednrb* expression did not show a decrease. Moreover, when treated with rosiglitazone in combination with GW9662, the expression levels of PPAR-γ appeared upregulated again, while the levels of *tyr*, *trp-2,* and *mitf* were all upregulated (Fig. S2b). In addition, the western-blot results also showed that rosiglitazone significantly increased the expression levels of *ppar-γ* and *tyr*. And the expression levels of *tyr*, a key factor of melanogenesis, decreased after GW9662 inhibited the expression of *ppar-γ* (Fig. S2c and d). Similar to the results of RT-PCR, the immunofluorescence results also showed that rosiglitazone increased the expression of PPAR-γ, TYR, MITF, EDNRB, and TRP-1/2. In contrast, GW9662 significantly inhibited the expression of PPAR-γ, TYR, MITF, EDNRB, and TRP-1/2. Moreover, the expression of TYR, MITF, EDNRB, and TRP-1/2 was elevated again when rosiglitazone and GW acted together on melanoma cells, as PPAR-γ was activated (Fig. 4c and d; Fig. S3a-d).

### Deficiency of PPAR Pathway Reduced Melanogenesis in Zebrafish

**Fig. 5:**
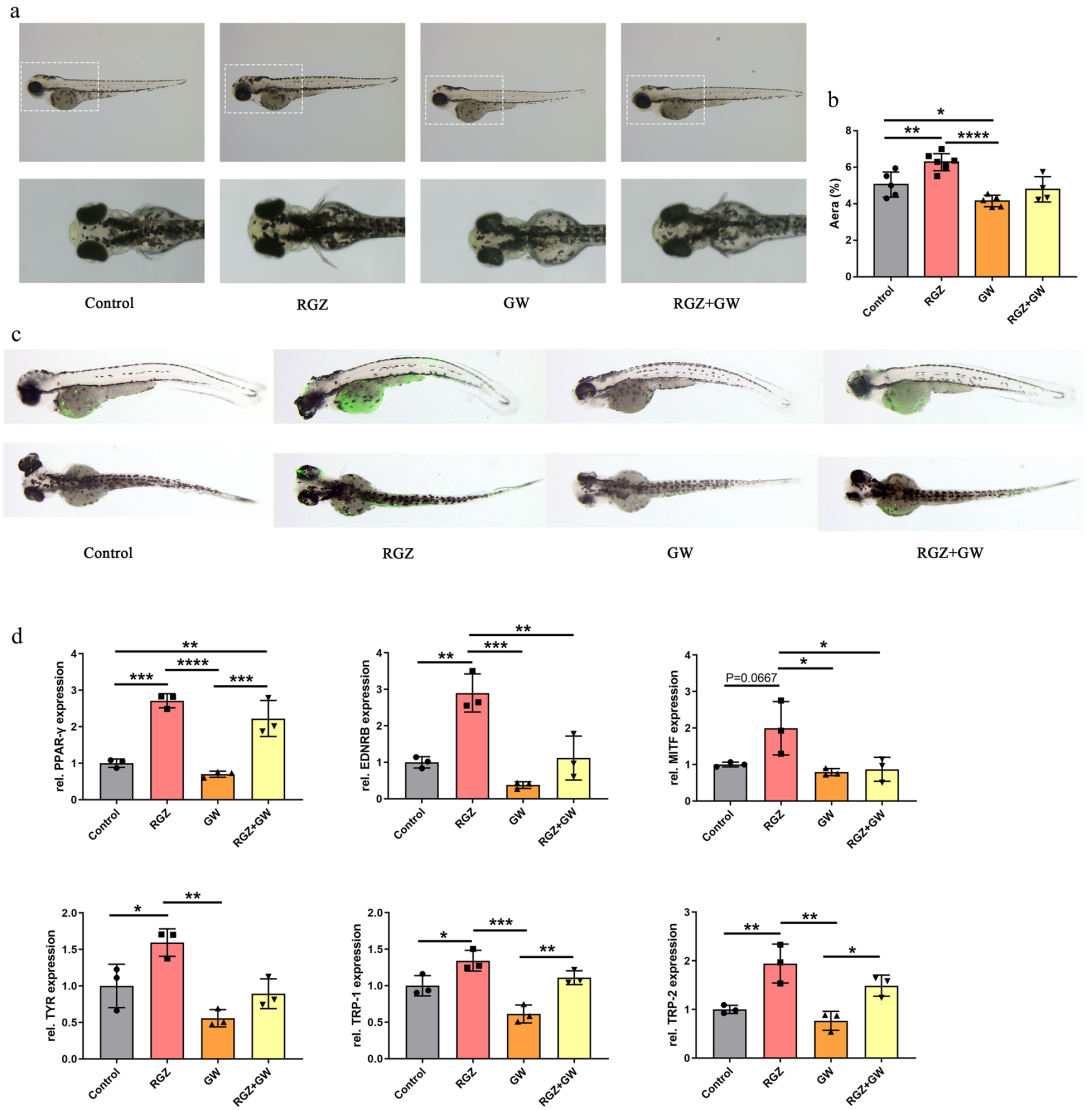
PAPR-γ pathway activation increases melanogenesis in zebrafish. **a** Melanin granules in the head of zebrafish and melanin granules of zebrafish at 72 hpf. **b** The area of melanin granules as a percentage of the head area was measured by ImageJ at 72 hpf. **c** Immunofluorescence analysis of zebrafish at 72 hpf after rosiglitazone and GW9662 treatment. **d** The expression of *ppar-γ*, *ednrb*, *mitf*, *tyr*, *trp-1*, and *trp-2* in zebrafish at 72 hpf. Data represent mean ± 95% confidence interval (CI); *p < 0.05; **p < 0.01; ***p < 0.001; *n* > 3.

To determine the effect of rosiglitazone on melanin synthesis *in vitro*, we treated zebrafish embryos with rosiglitazone at 24 hpf (1 h after fertilization) and assayed melanocyte content in the lateral body and head of zebrafish at 72 hpf. The results showed that rosiglitazone significantly increased melanin synthesis in zebrafish compared with the control group. And the melanin granules were significantly reduced after GW9662 treatment. However, no significant difference was found in melanin granules between the rosiglitazone + GW9662 group and the control group (*p* = 0.5893) (Fig. 5a and b).

In addition, the direct immunofluorescence results of zebrafish showed that the PPAR-γ expression was significantly higher in the rosiglitazone group than in the control group, while it was significantly lower in the GW9662 group. Moreover, the PPAR-γ protein fluorescence intensity of zebrafish showed a decrease after the co-treatment of rosiglitazone + GW9662 compared with the rosiglitazone group (Fig. 5c). These results suggest that rosiglitazone may promote pigmentation in zebrafish by up-regulating PPAR-γ expression. Further, we also examined the expression levels of PPAR-γ and black synthesis-related factors using RT-PCR. The results showed that rosiglitazone upregulated *ppar-γ* with a concomitant increase in the expression of *tyr*, *ednrb*, and *trp-1/2*, and conversely, when the expression level of *ppar-γ* was decreased, the expression levels of these melanin synthesis-related factors were also decreased (Fig. 5d).

## DISCUSSION

Vitiligo is the most common depigmented skin disease, and although the disease is not physically harmful or contagious, vitiligo is often psychologically devastating. The dysfunction or destruction of melanocytes, the main skin pigment-producing cells, plays an important role in the development of vitiligo [37, 38]. Increasing the understanding of inflammatory pathways in the pathogenesis of vitiligo and finding more therapeutic methods to promote the proliferation and function of melanocytes has been a hot topic in the field of vitiligo treatment.

In this study, we explored potential genes and signaling pathways associated with vitiligo and metabolic diseases by performing a comprehensive analysis of eligible vitiligo datasets from the GEO database. As expected, the expression of MC1R, TYR, TYRP1, and DCT was significantly downregulated during vitiligo pathogenesis. Among them, MC1R acts as a receptor for melanocytes and is able to regulate melanogenesis [39]. TYR, TYRP1, and DCT then constitute the enzyme system of melanosomes in melanocytes [40]. Activation of EDNRB increases melanin synthesis [41]. In addition, it is worth noting that the results of KEGG and GO analyses revealed important roles for pathways related to melanin synthesis, tyrosine metabolism, glycolysis, and inflammatory factors, such as “PPAR signaling pathway”, “tyrosine metabolism”, “nonalcoholic fatty liver disease (NAFLD) pathway”, “melanogenesis”, “IL-17 signaling pathway”, “glycolysis/gluconeogenesis”, and “galactose metabolism”. These results enrich the understanding of the pathogenesis of vitiligo.

Among the above pathways, we found that the PPAR pathway was significantly downregulated in vitiligo samples. Moreover, the results of gene-drug prediction also suggested that rosiglitazone, a specific agonist of PPAR-γ, might regulate the expression of ENDRB to enhance melanogenesis. Subsequently, the results of immunofluorescence staining showed that the expression levels of PPAR-γ, TYR, and EDNRB were significantly reduced in non-segmental vitiligo patients’ lesions skin along with the number of MITF-labeled melanocytes. Therefore, based on the above results, we have good reasons to believe that the PPAR-γ signaling pathway may regulate melanogenesis by altering the expression of ENDRB, and rosiglitazone, a specific agonist of PPAR-γ, may become a potential target for non-segmental vitiligo treatment.

Peroxisome proliferator-activated receptor signaling pathway (PPAR signaling pathway) is a nuclear hormone receptor activated by fatty acids and their derivatives, which regulates gene expression, cell proliferation, differentiation, apoptosis, inflammatory response, and tumorigenesis by binding to specific ligands. In particular, it plays an important role in lipid synthesis and glucose metabolism [42–44]. Interestingly, all three receptors of PPAR (PPAR-α, PPAR-β/δ, PPAR-γ) are expressed in human and rodent skin, which regulate a variety of skin-related functions, including keratinocyte proliferation, epidermal barrier maturation, wound healing, sebum secretion, and melanocyte proliferation [45–47]. Related studies have found that PPARs play an important role in the pathogenesis of many skin diseases, such as acne vulgaris [48], psoriasis [49], hirsutism [50], scleroderma [51], squamous cell carcinoma of the skin [52], and Kaposi’s sarcoma [53]. Among them, PPAR-γ is one of the hot spots of research. PPAR-γ receptor is mainly expressed in adipose tissue and has an important role in adipose metabolism and preadipocyte differentiation [54]. When PPAR-γ transcription factor is activated by lipid ligands or compound agonists, the differentiation and anti-inflammatory effects were promoted and cell proliferation is slowed [55]. Studies on PPAR-γ knockout mice have shown that PPAR-γ is crucial for maintaining normal insulin sensitivity, glucose metabolism, and lipid metabolism homeostasis and that its abnormal expression is closely associated with the development of type 2 diabetes, obesity, and cardiovascular disease [56, 57]. Interestingly, the prevalence of diabetes mellitus and insulin resistance is higher in vitiligo patients than in the non-vitiligo population [58, 59]. A large epidemiological survey by Afkhami-Ardekani et al. also noticed that the prevalence of comorbid vitiligo was 4.9% in 1100 patients with type 2 diabetes compared to 1.8% in the healthy population [60]. The PPARy pathway may be a potential pathway to explain the correlation between vitiligo and diabetes mellitus.

Recent studies have found that the PPAR family induces enhanced melanogenesis in melanocytes and melanoma cells by affecting the melanocyte-stimulating hormone receptor (MC1R) and melanocyte-associated transcription factor (MITF) signaling pathways [30]. Lee et al. [61] also reported that selegiline, the PPAR-γ agonist, increased human melanocyte and melanogenic capacity in cultured human skin. Moreover, selegiline also increased the migratory capacity of melanocytes, tyrosinase activity, and the expression level of MITF. Kang et al. [62] found that benzofibrate (activator of PZA-β/δ) had no significant effect on the melanin content of human melanocytes *in vitro*, but WY-14643 (activator of PPAR-α) and rosiglitazone (activator of PPAR-γ) inhibited melanocyte proliferation in a dose-dependent manner, and this growth inhibition is accompanied by activation of melanocytes, including an increase in the number of dendrites and an increase in cell volume. However, the PPAR-γ receptor and its agonist rosiglitazone are still less studied on melanogenesis, especially *in vitro* studies.

To further explore the potential therapeutic effects of PPAR-γ in non-segmental vitiligo, we investigated the potential effects of rosiglitazone, a specific agonist of PPAR-γ, on melanin synthesis according to the results of Gene-drug prediction. However, due to the difficulty in obtaining human primary melanocytes, melanogenesis-related studies are usually performed using pigment-producing melanoma cell lines. Therefore, a human-derived melanoma cell line (Mum-2C) was used for our next experiments [31]. The results for _L_-dopa showed that rosiglitazone increased tyrosinase activity in cells, whereas GW9662, a specific inhibitor of PPAR-γ, inhibited tyrosinase activity. Mechanistically, rosiglitazone activated the PPAR-γ pathway and upregulated the expression levels of EDNRB, TYR, and MITF, and conversely, when PPAR-γ was inhibited by GW9662, the expression of EDNRB, TYR, and MITF was significantly decreased. And, our results also showed that rosiglitazone effectively reversed the inhibitory effect of GW9662 on melanin synthesis. Notably, our *in viv*o study also revealed that the melanin synthesis capacity of zebrafish significantly increased with the upregulation of PPAR-γ expression and the expression levels of melanin synthesis-related factors such as TYR, EDNRB, and TRP-1/2 after rosiglitazone treatment. In contrast, after GW9662 inhibited the expression of PPAR-γ, melanin synthesis, and the expression of the above factors were suppressed in zebrafish.

In conclusion, PPAR-γ downregulation is closely related to cutaneous melanin loss, while rosiglitazone promotes melanogenesis by activating PPAR-γ and may be a potential drug for the treatment of non-segmental vitiligo.

### Supplementary Information

ESM 1 (DOCX 12060 kb)

## Abbreviations

BP: Biological processes
CC: Cellular component
DEGs: Differentially expressed genes
DMSO: Dimethyl sulfoxide
GEO: Gene Expression Omnibus
GO: Gene Ontology
hpf: Hour post-fertilization
FC: Fold change
KEGG: Kyoto Encyclopedia of Genes and Genomes
MCC: Maximal Clique Centrality
MF: Molecular function
MITF: Microphthalmia-associated transcription factor
PPAR: Peroxisome proliferator-activated receptor signaling pathway
PPI: protein–protein interaction
q-PCR: Quantitative polymerase chain reaction
TYR: Tyrosinase
