# Supplementary material for: Multiple gene-drug prediction tool reveals Rosiglitazone based treatment pathway for non-segmental vitiligo
Source: Inflammation. 2023 Dec 30;47(2):678–95. doi: 10.1007/s10753-023-01937-9 (PMC11074021; doi:10.1007/s10753-023-01937-9)
Supplement: Supplementary file 1 — (DOCX 12060 kb) [file 10753_2023_1937_MOESM1_ESM.docx]

| Patient id | Gender | Age | Duration | Progression | Classification | Biopsy Area^1^ | BSA^2^  ^(%)^ | Associated Disorders ^3^ or Family History |
| --- | --- | --- | --- | --- | --- | --- | --- | --- |
| 1  2  3  4  5  6  7  8  9  10 | F  F  M  M  F  F  F  M  F  M | 62Y  37Y  18Y  26Y  22Y  29Y  28Y  36Y  48Y  32Y | 2Y  1Y  3Y  2Y  5M  None  None  None  None  None | Stable  Stable  Stable  Stable  Unstable  None  None  None  None  None | NSV  NSV  NSV  NSV  NSV  Healthy volunteer  Healthy volunteer  Healthy volunteer  Healthy volunteer  Healthy volunteer | Vulva  Scalp  Face  Prepuce Limb  Cervical Back  Cervical Face  Back | 1  4  1.5  3  2  None  None  None  None  None | None  None  None  None  None  None  None  None  None  None |

Table SI. The Clinical Information of the Patients.

Notes:

^1^：lesional skin. The size of skin biopsy is about 1cm*1cm.

^2^: The severity of the disease is scored by body surface area (BSA) of depigmentation. ^3^: Associated disorders include psoriasis, alopecia areata, lupus erythematosus, rheumatoid arthritis, diabetes and so on. All patients had not received vitiligo-related treatment prior to surgical biopsy.

Abbreviations: F, female; M, male; Y, years; M, months; NSV, nonsegmental vitiligo; BSA, body surface area.

| Gene |  | Sequences ( 5′–3′) |
| --- | --- | --- |
| TYR  (cells) | Forward | GCAAAGCATACCATCAGCTCA |
|  | Reverse | GCAGTGCATCCATTGACACAT |
| TRP-1  (cells) | Forward | AGCCCTCAGTATCCCCATGAT |
|  | Reverse | CCCGGACAAAGTGGTTCTTTT |
| TRP-2  (cells) | Forward | AACTGCGAGCGGAAGAAACC |
|  | Reverse | CGTAGTCGGGGTGTACTCTCT |
| MITF  (cells) | Forward | GTGTCACTGATCCACTCCTTTC |
|  | Reverse | CCGTCTCTTCCATGCTCATAC |
| PPAR-γ  (cells) | Forward | ACCAAAGTGCAATCAAAGTGGA |
|  | Reverse | ATGAGGGAGTTGGAAGGCTCT |
| EDNRB  (cells) | Forward | GCAAACCGCAGAGATAATGACG |
|  | Reverse | GGACACAACCGTGTTGATGTATT |
| β-actin  (cells) | Forward | CCATCGTCCACCGCAAAT |
|  | Reverse | GCTGTCACCTTCACCGTTCC |
| TYR  (Zebrafish) | Forward | ACTACCGAGAGGCAGAGGTTCATC |
|  | Reverse | TTGGCGAACATTGGCGTGGAG |
| MITF  (Zebrafish) | Forward | TGTACAGCAATCATGCTCTTCC |
|  | Reverse | GTCCCCAGCTCCTTAATTCTGTC |
| PPAR-γ  (Zebrafish) | Forward | GACACGCACTCGCTGGACATC |
|  | Reverse | GGGTTGGGTCATTCTGTGTTGGG |
| β-actin  (Zebrafish) | Forward | GAAGGAGATCACCTCTCTTGCTC |
|  | Reverse | GTTCTGTTTAGAAGCACTTCCTGTG |

Table SII. Primer sequences for TYR, TRP-1, TRP-2, MITF, PPAR-γ and β-actin.

| Pathway | ID | Gene count | Log P-value | Genes |
| --- | --- | --- | --- | --- |
| Oxidative phosphorylation | hsa00190 | 24 | -12.1875 | ATP5F1C, ATP5F1D, ATP5F1E, ATP5PB, ATP5ME, ATP5PF, ATP6V0C, ATP5PO, COX6C, COX7A2, COX7B, NDUFA1, NDUFA4, NDUFB2, NDUFB3, NDUFB6, NDUFS4, UQCRB, ATP6V0D1, ATP6V1G1, ATP5MF, COX17, ATP5PD, NDUFA12 |
| Alzheimer's disease | hsa05010 | 20 | -6.91128 | APOE, ATP5F1C, ATP5F1D, ATP5F1E, ATP5PB, ATP5PF, ATP5PO, COX6C, COX7A2, COX7B, NDUFA1, NDUFA4, NDUFB2, NDUFB3, NDUFB6, NDUFS4, UQCRB, ATP5PD, BACE2, NDUFA12 |
| Parkinson's disease | hsa05012 | 18 | -6.81342 | ATP5F1C, ATP5F1D, ATP5F1E, ATP5PB, ATP5PF, ATP5PO, COX6C, COX7A2, COX7B, NDUFA1, NDUFA4, NDUFB2, NDUFB3, NDUFB6, NDUFS4, UQCRB, ATP5PD, NDUFA12 |
| Huntington's disease | hsa05016 | 20 | -6.05789 | ATP5F1C, ATP5F1D, ATP5F1E, ATP5PB, ATP5PF, ATP5PO, COX6C, COX7A2, COX7B, HDAC2, NDUFA1, NDUFA4, NDUFB2, NDUFB3, NDUFB6, NDUFS4, TFAM, UQCRB, ATP5PD, NDUFA12 |
| Non-alcoholic fatty liver disease (NAFLD) | hsa04932 | 12 | -2.87971 | COX6C, COX7A2, COX7B, MAP3K11, NDUFA1, NDUFA4, NDUFB2, NDUFB3, NDUFB6, NDUFS4, UQCRB, NDUFA12 |
| Ribosome | hsa03010 | 23 | -10.0209 | RPL7, RPL9, RPL24, RPL31, RPL34, RPL35A, RPL36AL, RPS7, RPS24, RPL14, MRPL33, MRPL13, MRPL22, MRPS18C, RPS27L, RPL26L1, MRPS17, MRPS21, MRPS15, MRPL36, MRPL32, MRPL1, MRPL21 |
| Spliceosome | hsa03040 | 15 | -5.10432 | CDC5L, SNRPA1, SNRPB2, SNRPD2, SNRPG, PRPF18, DDX46, THOC1, SLU7, LSM5, SYF2, LSM3, PPIL1, PRPF38B, RBM25 |
| Proteasome | hsa03050 | 8 | -4.41216 | PSMA2, PSMA3, PSMA4, PSMC6, PSMD1, SEM1, PSMD14, POMP |
| Nucleotide excision repair | hsa03420 | 8 | -4.2702 | CCNH, CDK7, ERCC5, PCNA, POLE2, RFC4, RPA3, GTF2H5 |
| DNA replication | hsa03030 | 6 | -3.27931 | PCNA, POLE2, PRIM1, RFC4, RPA3, SSBP1 |
| Mismatch repair | hsa03430 | 4 | -2.40547 | PCNA, RFC4, RPA3, SSBP1 |
| Basal transcription factors | hsa03022 | 7 | -3.54909 | CCNH, CDK7, GTF2A2, GTF2B, GTF2F2, TAF12, GTF2H5 |
| RNA degradation | hsa03018 | 9 | -3.43273 | PFKL, EXOSC9, MPHOSPH6, EXOSC8, MTREX, LSM5, LSM1, LSM3, ZCCHC7 |
| Homologous recombination | hsa03440 | 6 | -2.96898 | RAD51C, RBBP8, RPA3, SSBP1, SEM1, RAD50 |
| Galactose metabolism | hsa00052 | 5 | -2.74522 | GAA, PFKL, PGM2, AKR1B10, G6PC3 |
| Glycolysis / Gluconeogenesis | hsa00010 | 6 | -1.89568 | ADH7, PFKL, GAPDHS, PGM2, ACSS2, G6PC3 |
| Starch and sucrose metabolism | hsa00500 | 4 | -1.71028 | GAA, PYGB, PGM2, G6PC3 |
| Propanoate metabolism | hsa00640 | 5 | -2.68174 | ACADM, SUCLA2, HIBCH, ACSS2, MCEE |
| Carbon metabolism | hsa01200 | 8 | -1.75565 | ACADM, GPT, PFKL, SUCLA2, PGLS, HIBCH, ACSS2, MCEE |
| Valine, leucine and isoleucine degradation | hsa00280 | 4 | -1.30759 | ACADM, HMGCS2, HIBCH, MCEE |
| Melanogenesis | hsa04916 | 9 | -2.58537 | DCT, EDNRB, KIT, MC1R, KITLG, TYR, TYRP1, WNT5A, WNT7B |
| Tyrosine metabolism | hsa00350 | 4 | -1.75167 | ADH7, DCT, TYR, TYRP1 |
| Cell cycle | hsa04110 | 10 | -2.49361 | CCNA2, CCNH, CDK7, HDAC2, MAD2L1, PCNA, TTK, CCNE2, PTTG1, ANAPC10 |
| PPAR signaling pathway | hsa03320 | 7 | -2.32814 | ACADM, CD36, CYP27A1, DBI, FABP5, HMGCS2, SLC27A1 |
| Collecting duct acid secretion | hsa04966 | 4 | -2.1479 | ATP6V0C, CA2, ATP6V0D1, ATP6V1G1 |
| Phagosome | hsa04145 | 9 | -1.45675 | ATP6V0C, CD36, SCARB1, DYNC1I2, ITGAV, EEA1, ATP6V0D1, ATP6V1G1, TUBB2B |
| Vasopressin-regulated water reabsorption | hsa04962 | 5 | -2.07287 | ARHGDIA, DYNC1I2, VAMP2, DCTN6, DYNC2LI1 |
| RNA transport | hsa03013 | 11 | -1.95722 | PNN, TPR, EIF5B, THOC1, UPF2, NMD3, NUP107, UPF3B, UPF3A, NUP37, GEMIN6 |
| Nitrogen metabolism | hsa00910 | 3 | -1.91982 | CA2, CA9, CA14 |
| IL-17 signaling pathway | hsa04657 | 7 | -1.74293 | HSP90AA1, CXCL10, LCN2, MAPK6, PTGS2, S100A8, HSP90B1 |
| Pathways in cancer | hsa05200 | 19 | -1.66706 | CKS1B, CKS2, EDNRB, GNA11, HDAC2, HSP90AA1, ITGAV, KIT, KITLG, PTGS2, ELOC, TPR, HSP90B1, WNT5A, WNT7B, ZBTB16, CCNE2, ARNT2, LAMB4 |
| Small cell lung cancer | hsa05222 | 6 | -1.46162 | CKS1B, CKS2, ITGAV, PTGS2, CCNE2, LAMB4 |
| Lysosome | hsa04142 | 8 | -1.57967 | ATP6V0C, CTSD, CTSG, GAA, MAN2B1, SGSH, CTSF, ATP6V0D1 |
| ECM-receptor interaction | hsa04512 | 6 | -1.50582 | CD36, CHAD, HMMR, ITGAV, SDC4, LAMB4 |
| Ribosome biogenesis in eukaryotes | hsa03008 | 7 | -1.4885 | MPHOSPH10, GNL3, GNL2, NMD3, RIOK2, REXO1, WDR75 |
| Endocytosis | hsa04144 | 13 | -1.40339 | KIF5B, KIT, SNX2, EEA1, USP8, ARPC3, PIP5K1C, EPN1, WASHC3, CHMP5, VPS29, CHMP6, VPS37B |

Table SIII. Kyoto Encyclopedia of Genes and Genomes (KEGG) pathway analysis of integrated DEGs


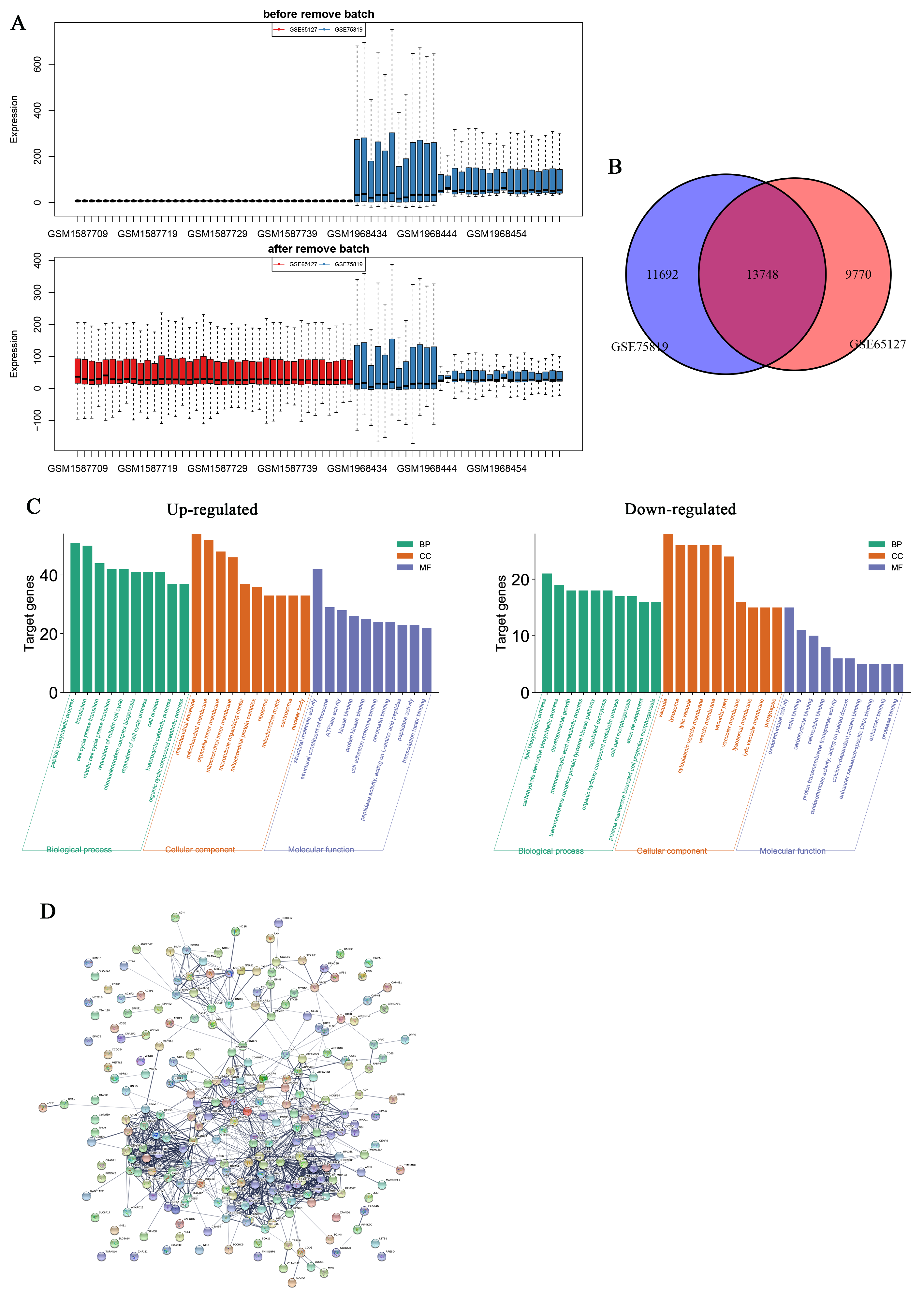


Figure S1. Processing of vitiligo dataset and functional enrichment results. (A) Batch effects elimination of datasets GSE65127 and GSE75819. (B) Venn plot revealed the number of overlapping genes among the three datasets. (C) For up/down regulated DEGs, the top 30 enriched GO terms sorted by adjusted P-value. (D) Protein-protein interaction (PPI) network of all DEGs.


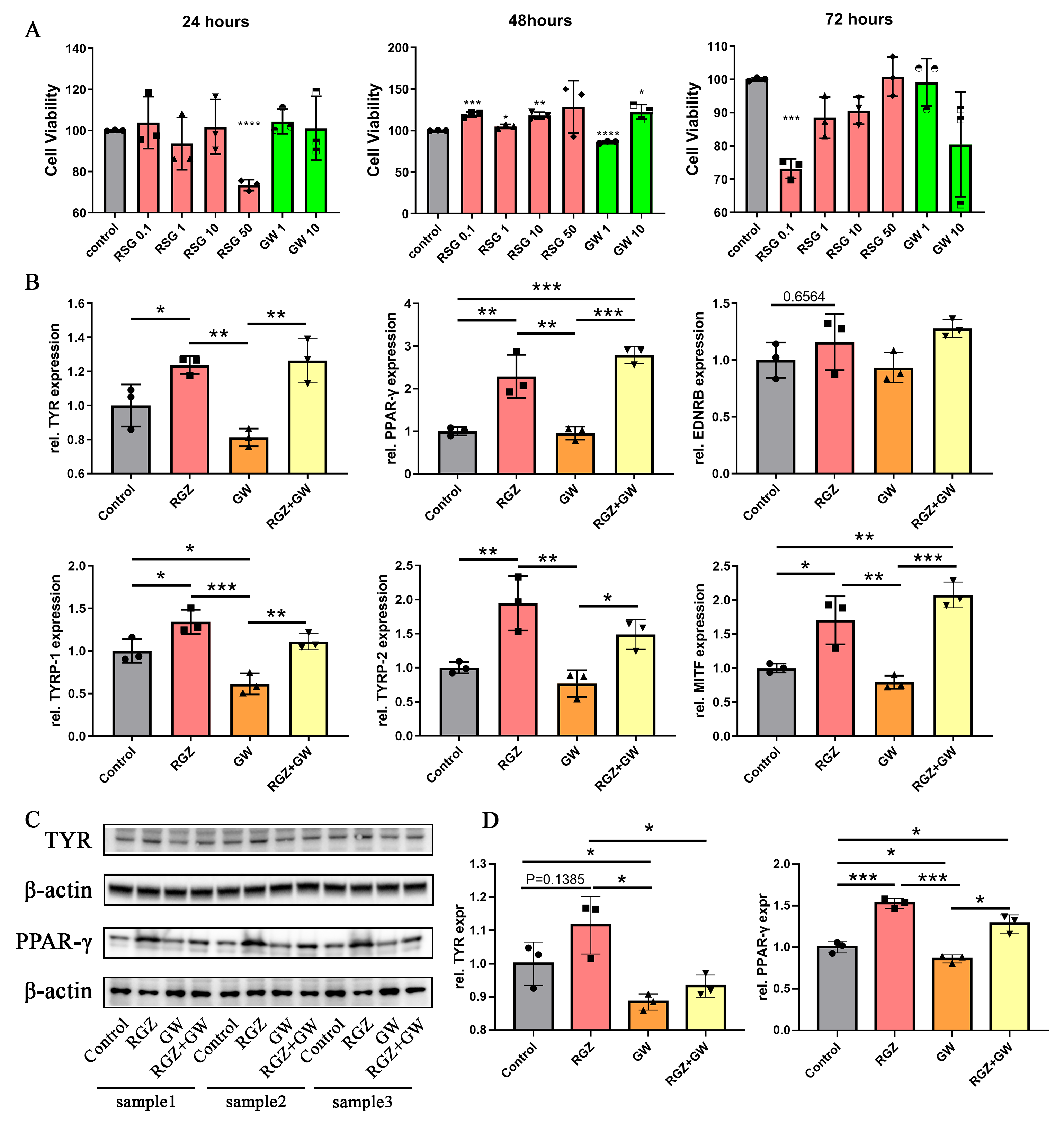


Figure S2. Supplementary data of the effect of PPAR-γ signaling pathway on melanogenesis. (A) Cell viability after treatment with rosiglitazone and GW9662 for 24, 48, 72 h. (B) The mRNA expression of *tyr*, *ppar-γ*, *ednrb*, *trp-1*, *trp-2* and *mitf* were analyzed by real-time PCR after treatment with rosiglitazone and GW9662 on 48 hours. (C) Western blotting was performed to analyze the expression of TYR and PPAR-γ after treated with rosiglitazone and GW9662. (D) The optical density of Western blotting. Data represent mean ± 95% confidence interval (CI) *P < 0.05, **P < 0.01, ***P < 0.001, and ****P < 0.0001, n = 3.


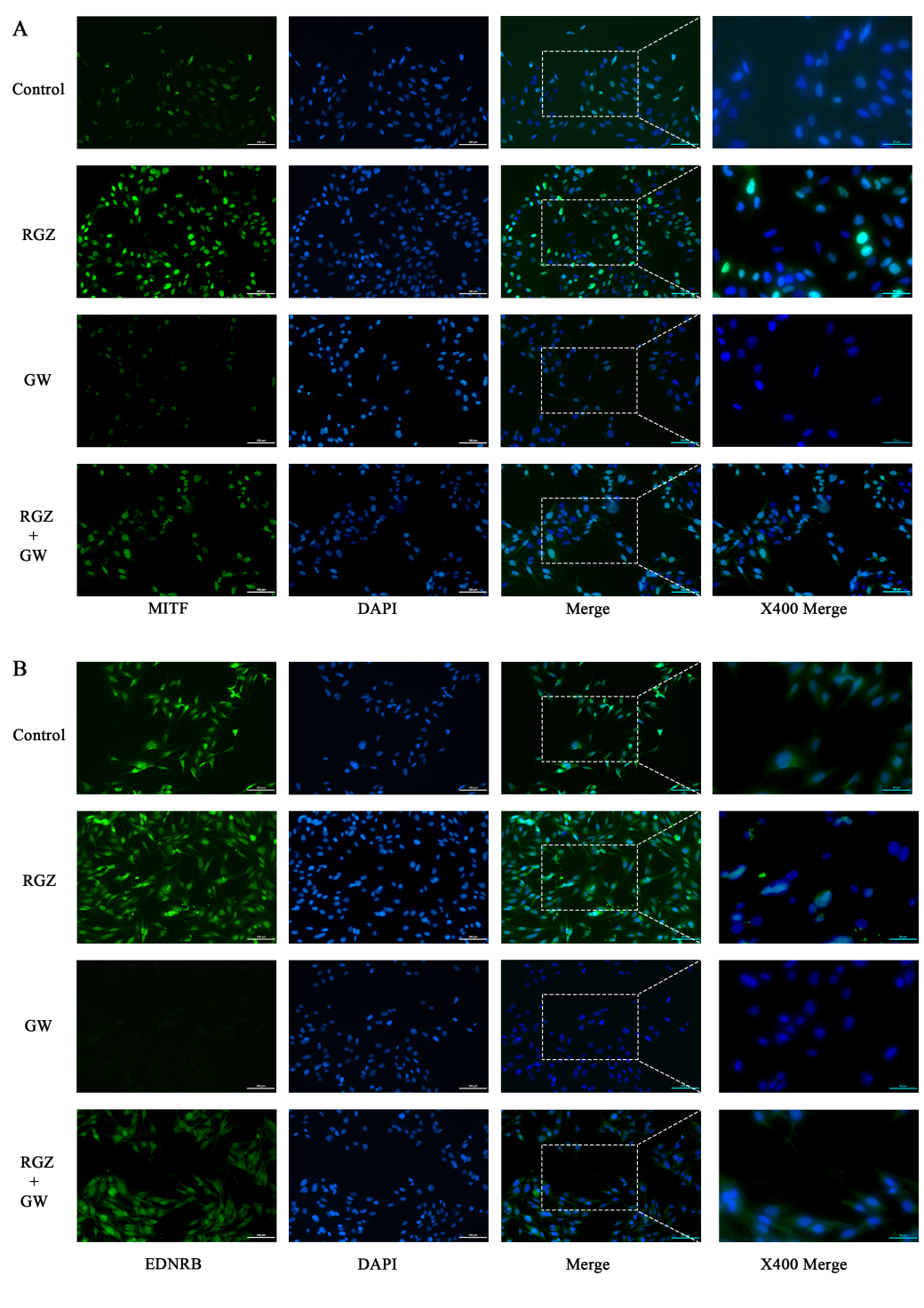


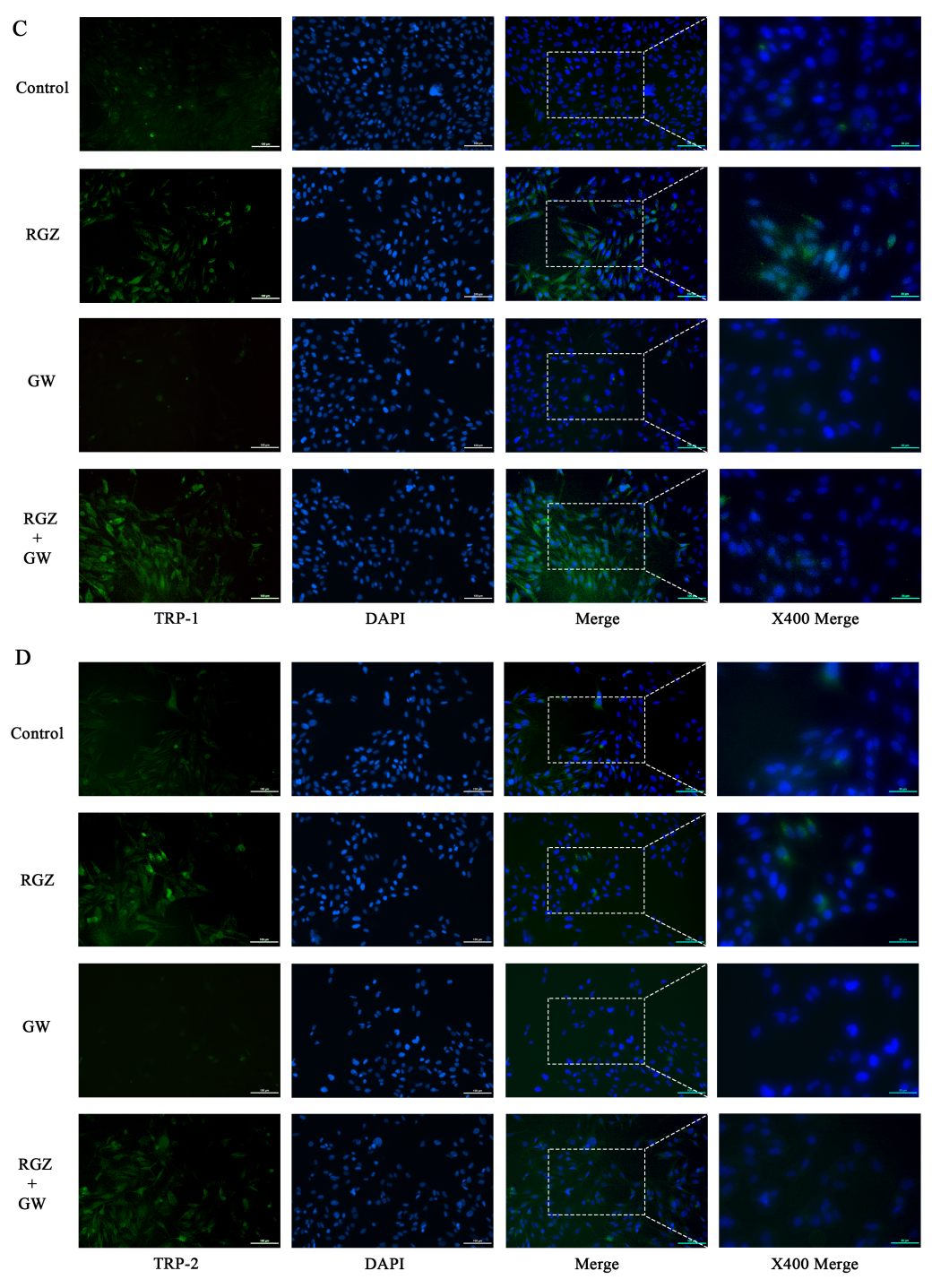


Figure S3. Effect of PPAR-γ signaling pathway on melanogenesis in melanoma cells (Mum-2C). (A-D) Immunofluorescence of MITF, EDNRB, TRP-1 and TRP-2 was shown after treatment with rosiglitazone and GW9662 for 48 hours. The antibody of MITF, EDNRB, TRP-1 and TRP-2 were stained with green, and the nuclei were counterstained with DAPI (blue).
